# Three Cases with Visual Hallucinations following Combined Ocular and Occipital Damage

**DOI:** 10.1155/2013/450725

**Published:** 2013-11-27

**Authors:** Bogusław Paradowski, Edyta Kowalczyk, Justyna Chojdak-Łukasiewicz, Aleksandra Loster-Niewińska, Monika Służewska-Niedźwiedź

**Affiliations:** Department of Neurology, Wroclaw Medical University, Ulica Borowska 213, 50-556 Wrocław, Poland

## Abstract

Charles Bonnet syndrome is an underrecognized disease that involves visual hallucinations in visually impaired patients. We present the cases of three patients who experienced complex visual hallucinations following various pathomechanisms. In two cases, diagnosis showed coexistence of occipital lobe damage with ocular damage, while in the third case it showed occipital lobe damage with retrobulbar optic neuritis. Theories of pathogenesis and the neuroanatomical basis of complex visual hallucinations are discussed and supported by literature review.

## 1. Introduction

Visual hallucinations may accompany many neurological and psychiatric disorders. There have been much controversy and disagreement regarding the diagnostic significance of ocular pathology, neurological disease, and the cognitive state in the occurrence of complex visual hallucinations. It was referred that stimulation of occipital lobe (area 17) leads to elementary hallucinations (e.g., photopsia), whereas that of occipitotemporal and occipitoparietal lobes (areas 18 and 19) was related to complex hallucinations (moving items, objects, and scenes) [1, 2]. The term pseudohallucinations means the involuntary experience (not a result of external stimuli) which is recognized by the person as unreal. Visual illusion is discrepancies between one's perception of an object or event and its actual state. Most frequently, visual hallucinations are regarded in Charles Bonnet syndrome, commonly induced by retinal disease, for example, age-related macular degeneration or other disturbances affecting the peripheral visual pathway. In 1760, Charles Bonnet first described in his essay how his 87-year-old grandfather, who had cataract surgery in both eyes, experienced spontaneous occurrence of formed visual hallucinations. Bonnet himself suffered from a painful loss of vision of unknown origin. During his retirement, he experienced visual hallucinations with many of the characteristics of the syndrome that bears his name. The term Charles Bonnet syndrome was coined in 1967 by George de Morsier [3]. Currently, detailed radiological examinations, such as single photon emission computed tomography (SPECT), are used to detect an asymmetrical perfusion in the temporal lobe, the parietooccipital region, and the brainstem in patients with Charles Bonnet syndrome [4].

## 2. Case Report

### 2.1. Case 1

**Figure 1:**
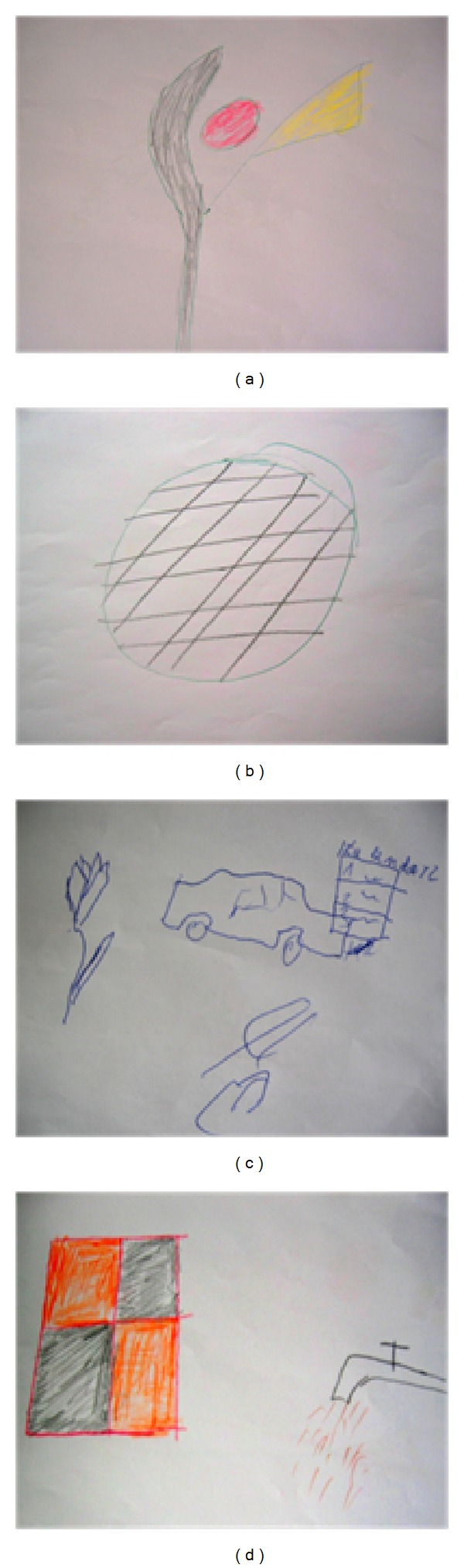
The drawings made by the patient (case 1) showing visual illusions ((a)-(b)) and visual pseudohallucinations ((c)-(d)).

A 65-year-old man with a prosthetic left eye (ocular trauma in childhood) underwent surgery of a proliferative process of the left lung (lobectomy with partial resection of the 2nd and 3rd ribs) with subsequent radiotherapy. Following the treatment, the patient reported throbbing headaches located in the temporal area and persisting for about three months. In addition, the patient had pseudohallucinations and visual illusions occurring for about 5 months. The illusions took the form of a ghost image, people with “second lips,” and “flower-like” and “lamp-like” images (Figures 1(a) and 1(b)).

On the other hand, pseudo-hallucinations were of human or animal shadows that moved over building rooftops and trams. They also had the form of letters appearing on walls, sweets, and flowers (Figures 1(c) and 1(d)). The patient also mentioned seeing an image of a square with smaller flashing squares inside it.

**Figure 2:**
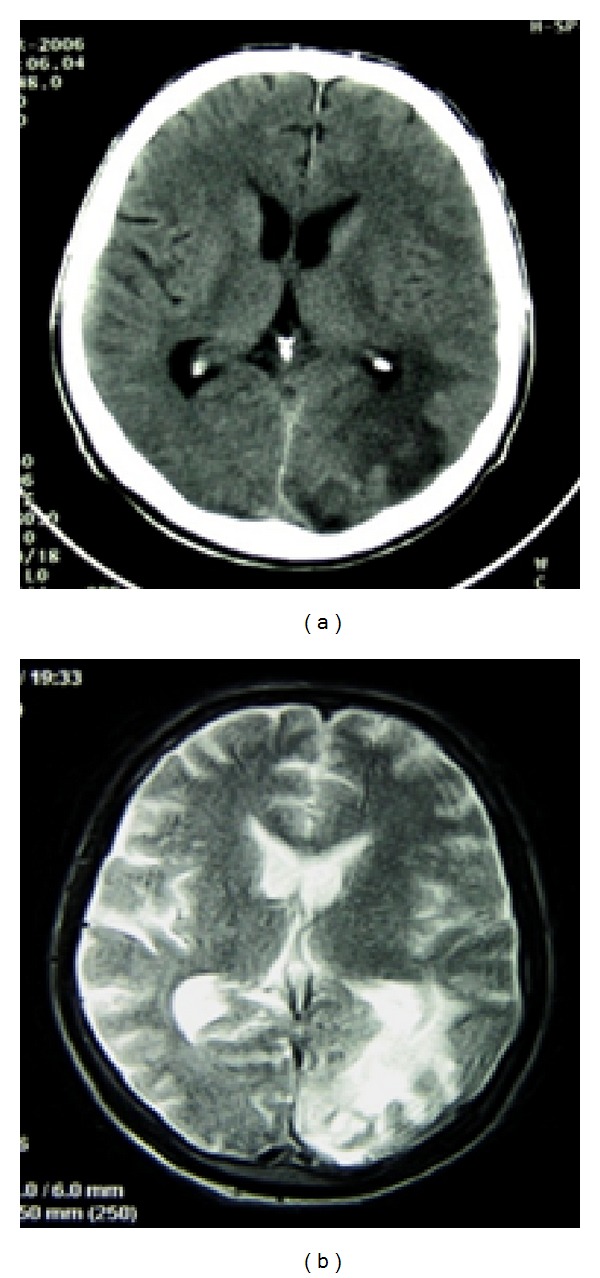
Metastatic changes in the left occipital region—a CT image (a) and an MR image (b).

Neurological examination revealed discrete paresis of the right hand, Babinski symptom, weakened knee reflexes, and no left ankle reflex. A CT scan of the head with administration of contrast medium and an MRI revealed a metastatic change in the left occipital irregular zone surrounded by an area of oedema of the white matter (Figures 2(a) and 2(b)). The patient was diagnosed with paracentral scotoma in the visual field related to the blind spot, located in the lower temporal quadrant, and lower sensitivity of the peripheral retina. The bottom of the cornea of the left eye was raised, resulting in blurred line vision. Visual evoked response testing showed prolonged latency and reduced amplitude of the left eye.

### 2.2. Case 2

**Figure 3:**
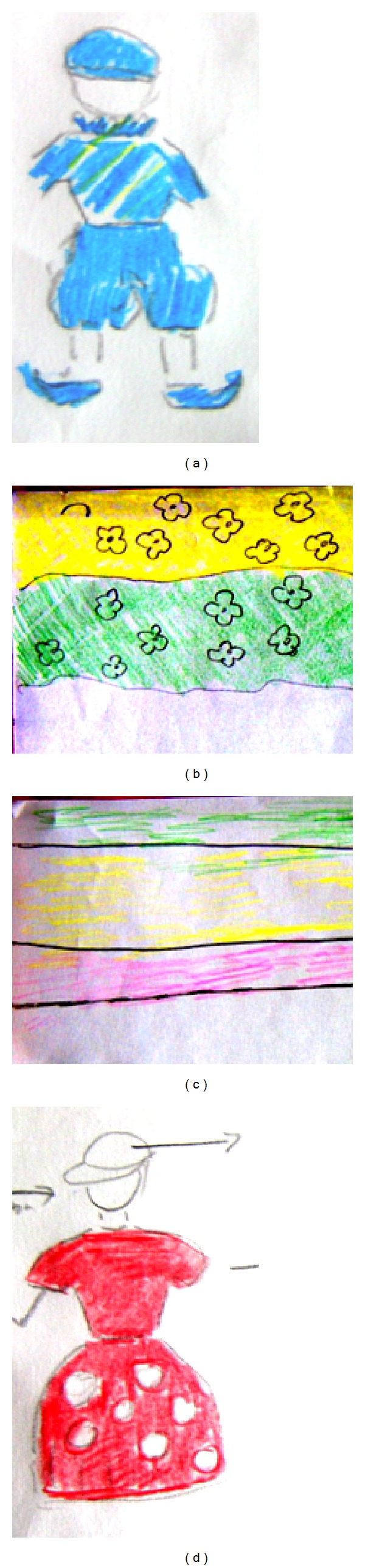
Drawings made by the patient (case 2) of visual illusions ((a) a real boy in illusioned blue dress) and simple ((b)-(c)) and complex ((d) unreal girl coming from a dream) visual pseudo-hallucinations.

An 84-year-old woman with a history of blindness of the right eye after cataract surgery was admitted to the Neurological Department due to visual disturbances. During the previous 2 months, she had reported seeing different people from her dreams, who seemed even more “real” when she was awake. Her condition leads to psychiatric consultation, which did not reveal any abnormalities. Two days prior to admission to our department, she started to experience simple colour phenomena. (She saw tricolour moving bands on a white wall.) On her way to hospital, she saw images of old-fashioned and vividly dressed children, a domed castle with a high tower, and nonexisting litter, boxes, and cases scattered in the street. She was able to give detailed descriptions of the clothes, colours, and movement of the items (Figures 3(a)–3(d)). She also suffered from hypertension and atrial fibrillation.

**Figure 4:**
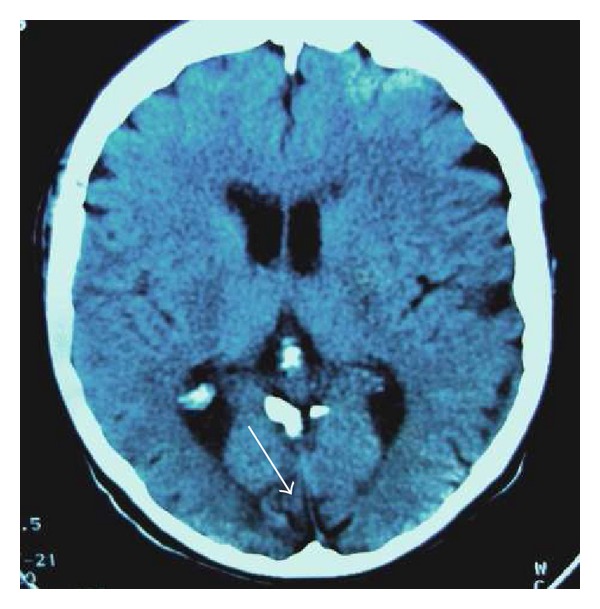
A CT image of the ischemic changes in the right occipital region.

Neurological examination revealed blindness of the right eye, impaired visual acuity of the left eye, and mild left hemiparesis. Her mental status did not reveal any psychotic symptoms; she had preserved her sense of direction and was self-aware and capable of reality testing. The patient scored 29 on the Minimental State Examination. The ophthalmologic examination of the left eye revealed a major impairment of the visual field (impaired paracentral vision in the lower quadrants) and macular degenerative changes. Computed tomography examination showed an ischaemic vascular lesion localized to the right median occipital lobe (Figure 4). Perfusion CT revealed necrosis of the medial part of the occipital lobe and hypoperfusion of the right interior occipital cortex. Neither neuroimaging method showed any other pathologic changes.

### 2.3. Case 3

**Figure 5:**
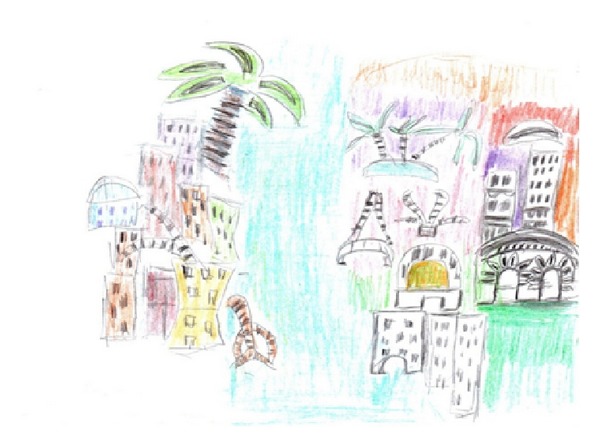
Drawings of the visual phenomena made by the patient (case 3).

**Figure 6:**
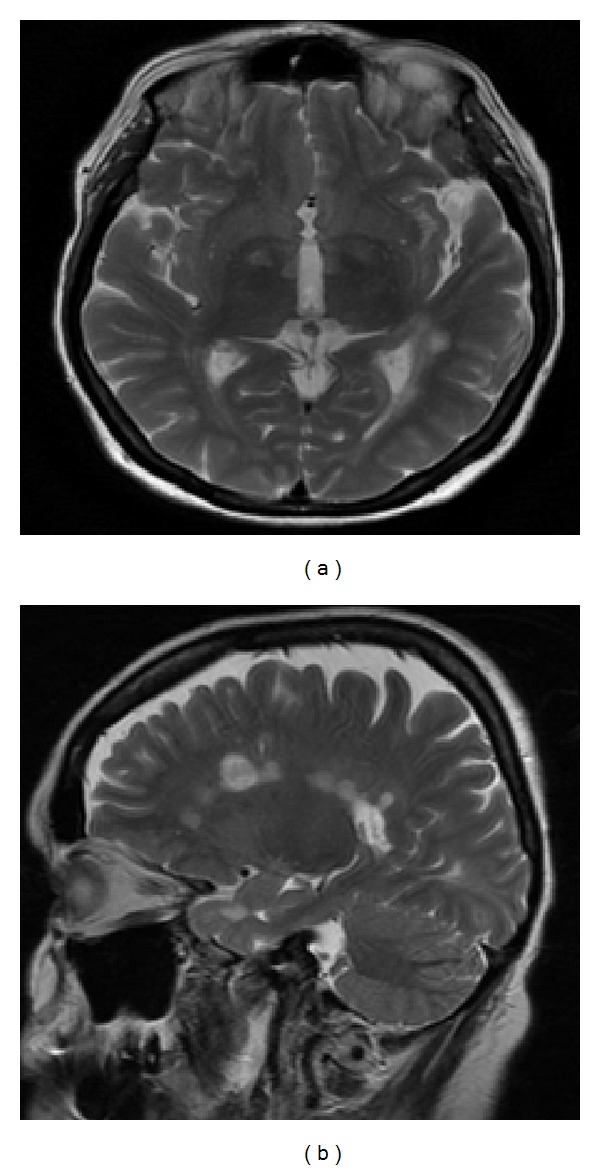
MR of the head; T2-weighted images (axial view (a) and sagittal view (b)) demonstrate numerous plaques in white matter distribution.

A 59-year-old Caucasian female, well known to our clinic, with established diagnosis of multiple sclerosis (MS), presented with a 10-day history of decreased right eye vision acuity. The patient also complained of visual phenomena appearing when she was observing objects with her left eye closed. The patient could see nonexistent patterns that she remembered from a piece of cloth she looked at in one of the shops (Figure 5). The visual sensations were not present when the patient was using her lefteye or both eyes. After a few days they disappeared. Her past medical history additionally consisted of hypothyreosis in the course of Hashimoto's disease, hypertension, gastroesophageal reflux disease, and hormone replacement therapy. An ophthalmologic assessment revealed Snellen visual acuity of 0.1 in the right eye and 0.8-0.9 in the left eye and partial loss of colour vision in the right eye. Her neurological examination demonstrated left-sided central facial palsy, mild right upper limb paresis with hyperactive reflexes in four limbs, and bilateral pyramidal signs. An auxiliary investigation showed bilaterally abnormal visual evoked potential latency (worse in the right eye) and single bursts of theta and sharp waves over the left hemisphere during photic stimulation in electroencephalography. Routine laboratory tests included full blood count, serum electrolytes, and glucose; kidney functions were within the normal range. Magnetic resonance imaging of the brain demonstrated multiple pathological areas consistent with demyelination, including lesions within the course of the optic radiation in the left hemisphere. None of these strengthened after an injection of gadolinium (Figures 6(a) and 6(b)). The patient was diagnosed with a relapse of MS with right-sided optic neuritis.

## 3. Discussion

The authors described the occurrence of visual phenomena of different aetiologies. There are numerous theories about the genesis of visual hallucinations. Asaad and Shapiro [5] identified three groups of potential causes of visual disturbances: psychophysiological (disruption of brain structure ex. seizure activity), psychobiochemical (specific biochemical and molecular changes), and psychodynamic. Teeple et al. [6] give a broad potential etiology of visual hallucinations such as psychosis, delirium, dementia, Anton's syndrome, seizures, migraines, peduncular hallucinosis, and sleep disturbances. In all three cases dementia, epilepsy, and other medical conditions and primary psychiatric illness as well as the effects of medications were ruled out. In the first and the second case the visual loss with lesion of the occipital cortex occurred a few years earlier and the visual phenomena lasted for 5 and 2 months. In the third patient right-sided retrobulbar optic neuritis preceded visual hallucination, which lasted a few days. Regardless of the location of an injury (optic nerve, visual pathway, visual cortex) and its pathogenesis (metastatic process, ischaemic or demyelinating), similar visual phenomena were formed. In the first case, pseudo-hallucinations and visual illusions occurred in the course of the loss of the left eye and focal metastasis in the left occipital lobe. In the second case, simple and complex visual pseudohallucinations arose from the blindness of an eye caused by cataract, a stroke in the necrotic area in the medial part of the right occipital lobe, and an ischemic zone around the back of the right cortex occipital lobe. In the third case, delusions and hallucinations occurred in the course of retrobulbar optic neuritis and optic pathway damage in the demyelinating process. In the first and third cases, the lesion was in left occipital lobe, in the second in right occipital area. The authors suggest that lateralization of the disruption of the visual pathways is not a significant factor in visual hallucinations. Manford and Andermann [7] described patients with visual hallucinations of different etiology (peduncular hallucinosis, Parkinson's disease, migraine, schizophrenia, epilepsy, and infarction)—in these cases the lesion was observed in left and right occipital lobe. The strongest risk factors for Charles Bonnet syndrome include bilateral visual impairment, declining visual acuity, cerebral damage, cognitive defects, social isolation, and sensory deprivation [8]. The reported cases demonstrate that the coexistence of an ocular injury and a small isolated lesion in the occipital lobe can lead to complex visual hallucinations. Perhaps hallucinations, as a disorder associated with organic perception affecting the central nervous system, may causally be linked to plasticity of the visual system. Another hypothesis suggests hyperexcitability of visual areas resulting from an imbalance between excitatory and inhibitory neural connections in the brain after damage [2, 8, 9].

Despite a number of hypotheses, the pathogenesis of visual hallucinations remains unclear. The synaptic architecture of large-scale networks and the manifestations of working memory, novelty-seeking behaviours, and mental imagery collectively help to loosen the rigid stimulus-response bonds that dominate the behaviour of lower animal species. Interconnected sets of transmodal nodes provide anatomical and computational epicentres for large-scale neurocognitive networks. Sensory information undergoes extensive associative elaboration and attentional modulation as it becomes incorporated into the texture of cognition. This process occurs along a core synaptic hierarchy which includes the primary sensory, upstream unimodal, downstream unimodal, heteromodal, paralimbic, and limbic zones of the cerebral cortex. The neuromatrix theory proposes the existence of large-scale neuronal networks capable of generating sensory phantoms [9].

The hallucinations are loosely associated with the cause and localization of a cerebral pathology but are strongly correlated with an abnormally activated neuronal network. The results of our cases suggest that multifocal damage in the visual system leads to an autonomous assembly of large-scale neuronal networks related to complex cognitive function.
